# Prevalence of metabolic syndrome and its associated risk factors among staffs in a Malaysian public university

**DOI:** 10.1038/s41598-021-87248-1

**Published:** 2021-04-14

**Authors:** Mohd Rizal Abdul Manaf, Azmawati Mohammed Nawi, Noorlaili Mohd Tauhid, Hanita Othman, Mohd Rizam Abdul Rahman, Hanizah Mohd Yusoff, Nazaruddin Safian, Pei Yuen Ng, Zahara Abdul Manaf, Nor Ba’yah Abdul Kadir, Kevina Yanasegaran, Siti Munirah Abdul Basir, Sowmya Ramakrishnappa, Kurubaran Ganasegeran

**Affiliations:** 1 Department of Community Health, Faculty of Medicine, Universiti Kebangsaan Malaysia, Kuala Lumpur, Malaysia; 2 Department of Family Medicine, Faculty of Medicine, Universiti Kebangsaan Malaysia, Kuala Lumpur, Malaysia; 3 Department of Pathology, Faculty of Medicine, Universiti Kebangsaan Malaysia, Kuala Lumpur, Malaysia; 4 Drug and Herbal Research Centre, Faculty of Pharmacy, Universiti Kebangsaan Malaysia, Kuala Lumpur, Malaysia; 5 Dietetic Program, Faculty of Health Sciences, Universiti Kebangsaan Malaysia, Kuala Lumpur, Malaysia; 6 Psychology Program, Faculty of Social Sciences and Humanities, Universiti Kebangsaan Malaysia, Kuala Lumpur, Malaysia; 7 Clinical Research Center, Seberang Jaya Hospital, Ministry of Health Malaysia, Penang, Malaysia

**Keywords:** Diseases, Endocrinology, Health occupations, Risk factors

## Abstract

Public health systems are concerned with the commensurate rise of metabolic syndrome (MetS) incidence across populations worldwide, due to its tendency to amplify greater risk of diabetes and cardiovascular diseases within communities. This study aimed to determine the prevalence of MetS and its associated risk factors among staffs in a Malaysian public university. A cross-sectional study was conducted among 538 staffs from the Universiti Kebangsaan Malaysia (UKM) between April and June 2019. MetS was defined according to JIS “Harmonized” criteria. A questionnaire that consisted of items on socio-demographics, lifestyle risk behaviors and personal medical history information was administered to participants. Subsequently, a series of physical examination and biochemical assessment was conducted at the hall or foyer of selected faculties in the university. Descriptive and inferential statistics were conducted using SPSS version 22.0. Multivariate models were yielded to determine the risk factors associated with MetS. Statistical significance was set at *P* < 0.05. The overall prevalence of MetS was 20.6%, with men having greater prevalence than women (24.9% vs. 18.3%). Prevalence of MetS increased with age. Factors contributed to MetS in the overall sample were BMI, hypertension, diabetes and physical activity of moderate intensity. Diabetes and hypertension were significantly associated with MetS in men, whereas BMI, diabetes and hyperlipidemia were significantly associated with MetS in women. Lifestyle behaviors and cardio-metabolic risk factors were associated with MetS for the overall sample, and across genders.

## Introduction

The burden of non-communicable diseases (NCDs) has caused substantial impact to health systems and economies worldwide. NCDs cause greater increase to morbidities and mortalities, reduced quality of life and escalated healthcare expenditures to governments, particularly in low- and middle-income countries (LMICs)^1,2^. Coupled with these unprecedented consequences of NCDs, global public health systems are being challenged with the rise of metabolic syndrome (MetS) incidence^3^. MetS constitutes a cluster of at least three out of five interrelated cardio-metabolic dysfunctions that occur concurrently^4–6^. The abnormalities include abdominal obesity, raised blood pressure, hyperglycemia, hypertriglyceridemia and low high-density lipoprotein (HDL) cholesterol levels^4–6^. These features predispose individuals to the development of diabetes and cardiovascular diseases^7^.

The global epidemic proportions of MetS was estimated to be around 20–25%^6^. When compared across regions, it was estimated that 12–37% of the Asian population were afflicted with MetS, while around 12–26% of the European population suffered the condition^8^. MetS affects approximately 25–40% of Malaysian adults, with its risks being elevated with advancing age^9^. The magnitude of MetS prevalence varies globally, especially in Asian countries as a result of differences in lifestyle behaviors and ethnicities^10^. As the proportion and distribution of body fat in Asians differed across populations in Europe or North American regions, it became fundamental to consider that the definition of obesity applied to Western populations cannot be adopted for Asian populations^11,12^. This could be observed with the rising trend of MetS prevalence reported in Singapore^13^, China^14^ and Malaysia^15^ when used the Asian adapted definitions on the National Cholesterol Education Program (NCEP)–Adult Treatment Panel III (ATP III) criteria. The Joint Interim Statement (JIS) “Harmonized” criteria definition that was later adopted was found to be more suitable to determine the proportions of MetS in Asian populations^16^.

While literature to determine MetS in populations was burgeoning rapidly, the exploration of such investigations to occupational groups were limited. Previous studies have identified that the prevalence of MetS among employees in a Taiwanese hospital was 12%^17^, among Chinese police officers was 23.2%^18^, among academic staffs in a Malaysian public university was 38.3%^19^ and among Malaysian government employees was 57.1%^20^. Literature has identified a multitude of factors to be associated with MetS. Demographic characteristics such as being a woman or older age was shown to escalate the risk of having MetS^21,22^, whereas lifestyle behaviors like physical activity, alcohol consumption, smoking, overweight or obesity^23–26^ were commonly linked to MetS across different geographies and populations. The current study was aimed to determine the prevalence of MetS and its associated risk factors using the JIS “Harmonized” criteria among staffs in a Malaysian public university.

## Methods

### Study design, setting and participants

This descriptive-analytical cross-sectional single-center study was conducted from April to June 2019 among staffs at the Universiti Kebangsaan Malaysia (UKM), Bangi, Selangor, Malaysia. Based on a sample-size calculation for a study of finite population^27^, with approximately 4000 employees at the Universiti Kebangsaan Malaysia (Bangi Campus), a minimum sample size of 522 staffs was calculated to represent a cross-section of the population and to allow the study to determine the prevalence of metabolic syndrome with a margin of error of ± 4%, as recommended by previous literature with a similar study population^28^. An additional 15% was included in the calculated sample to compensate for missing data and non-response^29^, for a final sample size of 600 staffs. A total of six hundred staffs from both academics and non-academics were randomly invited to participate in the study. Random selection was conducted using a free computer aided software (Research Randomizer)^30^. The sampling frame included the entire university’s staffs population, with their employment identity number provided by the Department of Registrar, Universiti Kebangsaan Malaysia. The single generated set of 600 random samples were identified, and subsequently, an invitation was sent out to the employees through their official email registered in the personal profile. The study was conducted at the hall or foyer of selected faculties in the university.

### Study inclusion and exclusion criteria

Permanent and contract staffs aged between 18 and 60 years old were included in the study. Staffs who were pregnant, breast-feeding and those who were on maternity leaves or sabbaticals were excluded.

### Ethics statement

This study complied with the guidelines convened in the Declaration of Helsinki. Ethical approval was obtained from the Universiti Kebangsaan Malaysia Research and Ethics Committee (approval number: UKM PPI.800-1/1/5/JEP-2019-391). Study objectives and benefits were explained verbally and in written form. Respondent’s confidentiality and anonymity were assured. Written consent was obtained from those who agreed to participate.

### Data collection and procedure

The study involved two stages. In stage one, respondents were required to complete a self-administered questionnaire that consisted of items on socio-demographic characteristics (gender and age), lifestyle risk behaviors (smoking status, alcohol consumption and physical activity level) and personal medical history (hypertension, diabetes and hyperlipidemia).

Smokers were defined as those who have smoked at least 100 cigarettes during their lifetime^31^. The item was assessed using a dichotomized response (yes/no). Alcohol consumption was assessed using a dichotomized response (yes/no), defined as monthly user consumption of alcoholic beverages, either wine, liquor or beer within the recent year^31^. Physical activity (PA) was assessed using the validated Malay version of the Global Physical Activity Questionnaire (GPAQ-M)^32^. The GPAQ-M comprises of 16 questions that asked participants about the intensity, frequency and duration of PA across 3 major domains, namely PA at work, PA during travel or transport and PA during recreation or leisure time, in addition, to an extra question that collected data on sedentary behavior and time, in minutes/day. A metabolic equivalent task (MET) value of 4 was designated as moderate intensity PA, while a value of 8 was assigned as vigorous intensity PA. These values of MET were subsequently multiplied by the number of days per week of PA and the duration on a typical day for each PA domain to tabulate the total PA (MET-minutes/week). The MET-minutes/week spent on each domain was subsequently computed to yield the overall PA level. High PA level was defined as vigorous-intensity activity on at least 3 days with at least 1500 MET-minutes/week or 7 days or more on any combinations of walking, moderate or vigorous intensity activities of at least 3000 MET-minutes/week. Moderate PA level was defined as 3 or more days of vigorous-intensity activity of at least 20 min/day or 5 or more days of moderate-intensity activity or walking of at least 30 min/day or 5 or more days of any combination of walking, moderate- or vigorous-intensity activities, that achieved a minimum of at least 600 MET-minutes/week. Participants who neither meet any of the previous two criteria were classified as having low PA level^33–35^. Personal medical history was based on respondents self-reported hypertension, diabetes or hyperlipidemia as diagnosed by a doctor or under current use of anti-hypertensives, anti-diabetics or lipid-lowering drugs.

In stage two, respondents were required to undergo a health screening session that constituted of general physical examination such as participant’s height, weight, body mass index (BMI), waist circumference (WC) and blood pressure (BP) measurements. Subsequently, medical laboratory tests for fasting blood glucose and lipid profiles were undertaken. Fasting blood (5 ml) was collected from each subject, separated using EDTA and serum separator tubes for biochemical investigations. Fasting plasma glucose (in mmol/L) was determined using the glucose oxidase method. Serum triglyceride (TG) (in mmol/L) was measured enzymatically after hydrolyzation of glycerol. High density lipoprotein cholesterol (HDL-c) (in mmol/L) was measured after precipitation of other lipoproteins with heparin manganese chloride mixture. Colorimetry/spectrophotometry for biochemistry assay and hexokinase for glucose were performed using Architect c16000 and c8000 (Abbott, Illinois, USA).

Height of the participant was measured barefooted by using a portable stadiometer (SECA, Germany) to the nearest 0.1 cm. Weight of the participant was measured using a digital lithium weighing scale (Tanita, Japan) calibrated to the nearest 0.1 kg, with the individual being dressed in light clothing and barefooted. The BMI was calculated by dividing body weight by the squared of height (kg/m^2^). BMI was later categorized based on the WHO BMI guideline (1998), which was also adopted in the National Health and Morbidity Survey (NHMS) 2015 Malaysia (< 25 kg/m^2^ as underweight to normal weight, 25.0 to 29.9 kg/m^2^ as overweight and ≥ 30 kg/m^2^ as obesity)^36^. To ease interpretations, the current study dichotomized the BMI category as < 30 kg/m^2^ (non-obese) and ≥ 30 kg/m^2^ (obese)^36^. WC was measured with participants wearing light clothing at mid-point between the lower rib margin and iliac crest using a flexible measuring tape to the nearest 0.1 cm. All anthropometric indices were measured twice and averaged to reduce measurement error as recommended by the International Standards for Anthropometric Assessment^37^. BP was measured twice on the same arm with a digital BP monitor (OMRON) after the individual had been seated at rest for at least 10 min. The systolic and diastolic BP measurements (in mmHg) were the mean of two readings.

### Criteria for MetS

This study adopted the Joint Interim Statement (JIS) “Harmonized” criteria for MetS as advocated by the International Diabetes Federation Task Force on Epidemiology and Prevention; National Heart, Lung, and Blood Institute; American Heart Association; World Heart Federation; International Atherosclerosis Society; and International Association for the Study of Obesity^6^. MetS was defined as having at least three of the following five risk factors: (1) raised waist circumference (WC) of ≥ 90 cm for men and ≥ 80 cm for women; (2) Raised serum triglycerides (TG) of ≥ 1.7 mmol/L; (3) Low high density lipoprotein cholesterol (HDL-c), defined as < 1.0 mmol/L for men and < 1.3 mmol/L for women; (4) Raised BP, defined as a systolic blood pressure (SBP) ≥ 130 mmHg or a diastolic blood pressure (DBP) ≥ 85 mmHg, or under current use of anti-hypertensive medications; and (5) Raised fasting blood sugar (hyperglycemia), defined as ≥ 5.6 mmol/L, or under current use of anti-diabetic medications.

### Statistical analysis

Analysis was conducted using IBM SPSS Statistics version 22.0^38^. Descriptive statistics were conducted for all variables in the study. Pearson chi-square test and binary logistic regressions were used to assess the associations between MetS and categorical independent variables such as demographics, lifestyle risk behaviors and personal medical history in this study. Crude odds ratios (cOR) were reported. Multiple logistic regression analysis using “Backward,” “Forward,” and “Enter” regression techniques were employed to determine the predictors of MetS in this sample. Adjusted odds ratios (aOR) were reported. Only the most parsimonious model that determined the factors associated with MetS for the overall sample and across both genders was selected. Multi-collinearity between independent variables was checked for the values of variance inflation factor (VIF) values not exceeding 10^39^. A relatively low VIF value (less than 5) confirms no interaction testing is warranted^40^. VIF values were yielded using the procedures recommended by IBM SPSS Statistics software guide^41^. Statistical significance was set at *P* < 0.05.

## Results

### Sample characteristics

Six hundred staffs were invited to participate and 538 consented to participate (participation rate: 89.7%). Of the total, 349 (64.9%) were women and 189 (35.1%) were men. The mean (SD) age of the participants was 43.4 (7.7) years and the age ranged between 27 and 60 years old. Most participants were aged between 35 and 44 years old, 309 (57.4%). Only thirty participants (7.4%) were smokers and six (1.5%) were alcohol drinkers, but the majority had a BMI < 30 kg/m^2^, 140 (74.1%). Most respondents reported to undertake high intensity PA, 226 (43.1%). Eighty-three (15.7%) of the participants had hypertension, 26 (4.9%) had diabetes and 74 (14%) had hyperlipidemia. The prevalence of MetS in this sample was 20.6% (Table 1).

**Table 1:** Sample characteristics (n = 538).

Characteristics	n (%)
***Demographics***	
**Sex**	
Men	189 (35.1)
Women	349 (64.9)
**Age group (years)**	
< 35	28 (5.2)
35–44	309 (57.4)
45–54	128 (23.8)
≥ 55	73 (13.6)
***Lifestyle risk factors***	
**Current smoker (n = 408)**	
No	378 (92.6)
Yes	30 (7.4)
**Alcohol drinker (n = 409)**	
No	403 (98.5)
Yes	6 (1.5)
**BMI**	
< 30	140 (74.1)
≥ 30	49 (25.9)
**Physical activity level (n = 524)**	
Low intensity	155 (29.6)
Moderate intensity	143 (27.3)
High intensity	226 (43.1)
***Medical conditions***	
**Hypertension (n = 527)**	
No	444 (84.3)
Yes	83 (15.7)
**Diabetes (n = 528)**	
No	502 (95.1)
Yes	26 (4.9)
**Hyperlipidemia (n = 529)**	
No	455 (86.0)
Yes	74 (14.0)
MetS*	
No	427 (79.4)
Yes	111 (20.6)

*MetS denotes metabolic syndrome.

### Prevalence of MetS and its components by age groups

The overall prevalence of MetS was 20.6% (24.9% in men and 18.3% in women). As exhibited in Table (2), this study showed a significantly higher prevalence of MetS in the older aged group for the overall sample and in women. The prevalence of MetS increased from 10.7 and 9.5% for those aged less than 35 years to 31.5% and 36.1% for those aged 55 years or more in the overall sample and in women respectively. Two MetS components (glucose and SBP) showed similar trends in both men and women. Both genders had the highest glucose and SBP levels at the age of 55 years or older. However, other MetS components (WC, TG and DBP) were significantly prevalent in women. The highest WC and TG levels were found among women aged 55 years or older, whereas raised DBP levels were mostly found in women between the ages 45–54 years (Table 2).

**Table 2:** Prevalence of MetS and its components by age groups.

Characteristics	Age group (years)				*P*-value
	< 35	35–44	45–54	≥ 55	
**Overall sample (n = 538)**					
MetS	3 (10.7)	51 (16.5)	34 (26.6)	23 (31.5)	0.005
**Men (n = 189)**					
MetS	1 (14.3)	20 (20.4)	16 (34.0)	10 (27.0)	0.299
Raised WC	4 (57.1)	55 (56.1)	30 (63.8)	24 (64.9)	0.733
Raised TG	2 (28.6)	30 (30.6)	18 (38.3)	7 (18.9)	0.294
Low HDL-c	1 (14.3)	11 (11.2)	10 (21.3)	4 (10.8)	0.385
Raised glucose	0 (0.0)	11 (11.2)	11 (23.4)	12 (32.4)	0.013
Raised SBP	2 (28.6)	27 (27.6)	20 (42.6)	24 (64.9)	0.001
Raised DBP	2 (28.6)	36 (36.7)	26 (55.3)	20 (54.1)	0.082
**Women (n = 349)**					
MetS	2 (9.5)	31 (14.7)	18 (22.2)	13 (36.1)	0.010
Raised WC	9 (42.9)	121 (57.3)	54 (66.7)	28 (77.8)	0.024
Raised TG	0 (0.0)	19 (9.0)	12 (14.8)	10 (27.8)	0.003
Low HDL-c	8 (38.1)	58 (27.5)	17 (21.0)	9 (25.0)	0.413
Raised glucose	1 (4.8)	16 (7.6)	16 (19.8)	9 (25.0)	0.002
Raised SBP	4 (19.0)	36 (17.1)	35 (43.2)	24 (66.7)	< 0.001
Raised DBP	4 (19.0)	58 (27.5)	35 (43.2)	13 (36.1)	0.035

### Risk factors associated with MetS by binary logistic regression

MetS for the overall sample was significantly higher among alcohol drinkers (cOR 8.2, 95% CI 1.4–45), those with BMI ≥ 30 kg/m^2^ (cOR 3.5, 95% CI 2.2–5.4), those who practice moderate intensity PA (cOR 1.8, 95% CI 1.1–3.3), and among those with hypertension (cOR 3.9, 95% CI 2.3–6.4), diabetes (cOR 5.1, 95% CI 2.3–11.3) or hyperlipidemia (cOR 1.8, 95% CI 1.1–3.1). In men, MetS was significantly higher among those with BMI ≥ 30 kg/m^2^ (cOR 2.2, 95% CI 1.1–4.5), and among those having hypertension (cOR 3.4, 95% CI 1.6–7.1) or diabetes (cOR 4.6, 95% CI 1.6–13.1). In women, MetS was significantly higher among alcohol drinkers (cOR 9.4, 95% CI 1.2–100.0), those with BMI ≥ 30 kg/m^2^ (cOR 4.7, 95% CI 2.7–8.3), and those having hypertension (cOR 4.1, 95% CI 2.1–8.1), diabetes (cOR 4.8, 95% CI 1.3–16.7) or hyperlipidemia (cOR 3.3, 95% CI 1.5–7.4) respectively. These associations were statistically significant (Table 3).

**Table 3:** Risk factors associated with MetS by Pearson chi-square and binary logistic regressions.

Risk factors	MetS in overall samples (n = 538)		cOR (95% CI)	MetS in Men (n = 189)		cOR (95% CI)	MetS in Women (n = 349)		cOR (95% CI)
	Yes n (%)	No n (%)		Yes n (%)	No n (%)		Yes n (%)	No n (%)	
**Sex**									
Men	47 (24.9)	142 (75.1)	1.5 (0.9–2.2)	–	–	–	–	–	–
Women	64 (18.3)	285 (81.7)	1	–	–	–	–	–	–
**Current smoker**									
No	75 (19.8)	303 (80.2)	1	28 (23.5)	91 (76.5)	1	47 (18.1)	212 (81.9)	#
Yes	7 (23.3)	23 (76.7)	1.2 (0.5–3.3)	7 (24.1)	22 (75.9)	1.1 (0.4–2.5)	0 (0.0)	1 (100.0)	#
**Alcohol drinker**									
No	79 (19.6)	324 (80.4)	1	34 (23.3)	112 (76.7)	1	45 (17.5)	212 (82.5)	1
Yes	4 (66.7)	2 (33.3)	8.2 (1.4–45.0)**	2 (66.7)	1 (33.3)	6.5 (0.6–76.0)	2 (66.7)	1 (33.3)	9.4 (1.2–100.0)*
**BMI**									
< 30	58 (14.6)	338 (85.4)	1	29 (20.7)	111 (79.3)	1	29 (11.3)	227 (88.7)	1
≥ 30	53 (37.3)	89 (62.7)	3.5 (2.2–5.4)***	18 (36.7)	31 (63.3)	2.2 (1.1–4.5)**	35 (37.6)	58 (62.4)	4.7 (2.7–8.3)***
**Physical activity intensity level**									
Low	24 (15.5)	131 (84.5)	1	9 (20.5)	35 (79.5)	1	15 (13.5)	96 (86.5)	1
Moderate	36 (25.2)	107 (74.8)	1.8 (1.1–3.3)**	15 (33.3)	30 (66.7)	1.9 (0.7–5.1)	21 (21.4)	77 (78.6)	1.7 (0.8–3.6)
High	48 (21.2)	178 (78.8)	1.5 (0.9–2.5)	23 (23.5)	75 (76.5)	1.2 (0.5–2.8)	25 (19.5)	103 (80.5)	1.5 (0.8–3.1)
**Hypertension**									
No	73 (16.4)	371 (83.6)	1	28 (19.4)	116 (80.6)	1	45 (15.0)	255 (85.0)	1
Yes	36 (43.4)	47 (56.6)	3.9 (2.3–6.4)***	18 (45.0)	22 (55.0)	3.4 (1.6–7.1)**	18 (41.9)	25 (58.1)	4.1 (2.1–8.1)***
**Diabetes**									
No	94 (18.7)	408 (81.3)	1	37 (21.9)	132 (78.1)	1	57 (17.1)	276 (82.9)	1
Yes	14 (53.8)	12 (46.2)	5.1 (2.3–11.3)***	9 (56.3)	7 (43.8)	4.6 (1.6–13.1)**	5 (50.0)	5 (50.0)	4.8 (1.3–16.7)**
**Hyperlipidemia**									
No	87 (19.1)	368 (80.9)	1	35 (25.2)	104 (74.8)	1	52 (16.5)	264 (83.5)	1
Yes	22 (29.7)	52 (70.3)	1.8 (1.1–3.1)**	11 (23.9)	35 (76.1)	0.9 (0.4–1.2)	11 (39.3)	17 (60.7)	3.3 (1.5–7.4)**

*Statistical significance (*P* < 0.05). **Statistical significance (*P* < 0.005). ***Statistical significance (*P* < 0.001). cOR (crude odds ratio). #cOR could not be yielded as one cell contained zero count during crosstabs.

### Risk factors associated with MetS by multiple logistic regression analyses

All statistically significant risk factors associated with MetS in the univariate analyses were included in the multivariate analyses. For the overall sample, the multivariable model had four statistically significant risk factors associated with MetS: BMI ≥ 30 kg/m^2^ (aOR 3.1, 95% CI 1.8–5.5; *P* < 0.001), moderate intensity PA (aOR 2.5, 95% CI 1.2–5.0; *P* = 0.015), having hypertension (aOR 2.0, 95% CI 1.1–4.3; *P* = 0.023) and diabetes (aOR 3.9, 95% CI 1.3–11.4; *P* = 0.011). The total model for the overall sample was significant and accounted for 19% of the variance (Table 4). In men, the multivariable model retained two statistically significant risk factors associated with MetS: having hypertension (aOR 2.4, 95% CI 1.1–5.4; *P* = 0.029) and diabetes (aOR 3.8, 95% CI 1.3–11.1; *P* = 0.031). The total model was significant and accounted for 15% of the variance (Table 5). For women, three risk factors associated with MetS were retained in the multivariable model: BMI ≥ 30 kg/m^2^ (aOR 4.6, 95% CI 2.3–8.2; *P* < 0.001), having diabetes (aOR 4.2, 95% CI 1.4–9.2; *P* = 0.023) and hyperlipidemia (aOR 3.2, 95% CI 1.1–6.0; *P* = 0.030). The total model was significant and accounted for 18% of the variance (Table 6). There was no multi-collinearity between independent variables in all three models, hence interaction analysis was not warranted. In all three multivariable analyses, the “Backward Wald” technique yielded the most parsimonious models.

**Table 4:** Risk factors associated with MetS in overall samples (adjusted for age) by multiple logistic regression (Backward Wald).

Risk factors	*B*	SE	Wald	Exp (*B*)	95% CI	*P*-value	VIF
**Alcohol drinker**							
No	Ref	Ref	Ref	Ref	Ref	Ref	1.037
Yes	− 1.7	0.9	2.8	5.3	0.7–33.0	0.095	
**BMI**							
< 30	Ref	Ref	Ref	Ref	Ref	Ref	1.056
≥ 30	− 1.1	0.3	15.6	3.1	1.8–5.5	< 0.001	
**Physical activity intensity level**							
Low	Ref	Ref	Ref	Ref	Ref	Ref	1.004
Moderate	− 0.9	0.4	6.3	2.5	1.2–5.0	0.015	
High	− 0.5	0.3	2.3	1.7	0.8–3.3	0.126	
**Hypertension**							
No	Ref	Ref	Ref	Ref	Ref	Ref	1.133
Yes	− 0.7	0.3	4.1	2.0	1.1–4.3	0.023	
**Diabetes**							
No	Ref	Ref	Ref	Ref	Ref	Ref	1.048
Yes	− 1.3	0.5	6.5	3.9	1.3–11.4	0.011	

Exp(*B*) gives adjusted odds ratio (aOR). VIF (Variance Inflation Factor). Ref (Reference category).

**Table 5:** Risk factors associated with MetS in men (adjusted for age) by multiple logistic regression (Backward Wald).

Risk factors	*B*	SE	Wald	Exp (*B*)	95% CI	*P*-value	VIF
**BMI**							
< 30	Ref	Ref	Ref	Ref	Ref	Ref	1.062
≥ 30	− 0.7	0.4	3.6	2.1	0.9–4.5	0.058	
**Hypertension**							
No	Ref	Ref	Ref	Ref	Ref	Ref	1.112
Yes	− 0.9	0.4	4.8	2.4	1.1–5.4	0.029	
**Diabetes**							
No	Ref	Ref	Ref	Ref	Ref	Ref	1.050
Yes	− 1.3	0.6	5.6	3.8	1.3–11.1	0.031	

Exp(*B*) gives the adjusted odds ratio (aOR). VIF (Variance Inflation Factor). Ref (Reference category).

**Table 6:** Risk factors associated with MetS in women (adjusted for age) by multiple logistic regression (Backward Wald).

Risk factors	*B*	SE	Wald	Exp (*B*)	95% CI	*P*-value	VIF
**BMI**							
< 30	Ref	Ref	Ref	Ref	Ref	Ref	1.081
≥ 30	− 1.5	0.4	18.5	4.6	2.3–8.2	< 0.001	
**Diabetes**							
No	Ref	Ref	Ref	Ref	Ref	Ref	1.042
Yes	− 2.2	0.9	5.2	4.2	1.4–9.2	0.023	
**Hyperlipidemia**							
No	Ref	Ref	Ref	Ref	Ref	Ref	1.114
Yes	− 1.2	0.6	4.7	3.2	1.1–6.0	0.030	

Exp(*B*) gives the adjusted odds ratio (aOR). VIF (Variance Inflation Factor). Ref (Reference category).

## Discussion

### Core summary findings

This study aimed to determine the prevalence of MetS and its associated risk factors among staffs in a Malaysian public university. The overall prevalence rate of MetS in this sample was 20.6%. Prevalence rate exhibited a commensurate rise with age, and was significantly higher in older aged people for the overall sample and in women, however, for men, the prevalence rate trended a non-significant S-shaped pattern with increasing age. Factors significantly associated with MetS in the overall sample were BMI ≥ 30 kg/m^2^, hypertension, diabetes and physical activity of moderate intensity. Gender-specific-risks regression models found that diabetes was the most important factor to be significantly associated with MetS in men (Wald value = 5.6), while a BMI ≥ 30 kg/m^2^ was the most substantial factor to be significantly associated with MetS in women (Wald value = 18.5).

### Comparison with existing literature

#### Prevalence of MetS and its components

This study found that the overall prevalence of MetS was 20.6%. The prevalence rate reported in this study was higher than that found in Taiwanese high-tech industry workers (8.2%)^42^, the Philippine general population (18.6%)^43^ and a rural Ugandan adult cohort (19.1%)^44^, but lower than that found in populations across Iran (ranged between 33.1% and 37.1%)^26,45^, Brazil (34.1%)^24^, Indonesia and the Netherlands (39% vs. 29.2%)^46^, China (ranged between 24.2 and 42.6%)^23,25,31^, Canada (25%)^47^ and Australia (ranged between 21.1 and 30.7%)^48^. From the Malaysian context, population-based prevalence estimates for MetS ranged between 25 and 40%^9,16,49^, while for specific sub-groups, the prevalence rates of MetS for patients with type 2 diabetes mellitus ranged between 73 and 85%^50^, among elderly people was 43.4%^51^, non-diabetic women post gestational diabetes mellitus was 22%^52^, and for vegetarians accounted for approximately 24.2%^53^. The bulk of existing literature reported that the prevalence of MetS was significantly higher in women than men^26,43,54–57^. In contrary to those findings, the current study showed that the prevalence of MetS in men was higher than in women (24.9% vs. 18.3%) and that no statistically significant difference was observed. This result was consistent with emerging works from the Indian^58^ and Chinese^25,59^ cohorts that evaluated prevalence rates across genders. The stratified analysis by age showed some interesting epidemiological observations in this study. Men aged < 55 years of age had higher prevalence of MetS than in women within the same aged group, but this observation was not statistically significant. However, a reversed phenomenon occurred for those aged ≥ 55 years of age, with women exhibiting greater prevalence rate of MetS than men, and this association was statistically significant. Similar observation was found in a previous study^59^. The current study found higher prevalence of MetS components in women than men. Two components (raised glucose and SBP) showed a significant upward linear trend with increasing age in both genders. However, other statistically significant MetS components (raised WC, TG and DBP) were only prevalent in women. These findings were inconsistent with previous studies^26,31^.

Investigators to-date often struggled to explain the controversial linkages between MetS with age- and gender-specific associations. The complexities surrounding the variation of prevalence rates across different study populations, regions, countries and settings were difficult to decipher and should be interpreted with caution. Four plausible attributes could be advocated to explain probable associations. These include methodological, environmental, hormonal, and lifestyle influences on the burden of MetS across populations. From the methodological domain, a prominent factor leading to variation of prevalence rates across studies could be attributed to different criteria and operational definitions applied to diagnose MetS. Four distinctive criteria were used across the scientific literature to determine the diagnosis and epidemiology of MetS till date, namely the World Health Organization (WHO)^60^, the National Cholesterol Education Program (NCEP)—Adult Treatment Panel III (ATP III)^4,61^, the International Diabetes Federation (IDF)^62^ and the Joint Interim Statement (JIS) “Harmonized”^6^ definitions. As the proportion and composition of body fat in Asians differed to European populations, it became apparent that the JIS Harmonized criteria was more suitable to determine the risk of MetS for populations in Asia, given its pre-defined cut-offs for central obesity (waist circumference for men ≥ 90 cm and women ≥ 80 cm) and reduced cut-off for hyperglycemia (≥ 5.6 mmol/L instead of 6.1 mmol in the NCEP-ATP III criteria). This was evident as Asian studies that used JIS Harmonized criteria to diagnose MetS showed distinctive variations when compared to other definitions^13–15,63^. From the environmental perspective, it was hypothesized that post-exposure serum perfluoroalkyl chemicals (PFCs) that are widely used in consumer products manufacture distorts glucose homeostasis and influence gender-specific MetS indicators^64,65^. Hormonal factors such as postmenopausal weight gain and confounded risk profiles might have accounted higher prevalence rate of MetS in women than men^51^. This postulation may be somewhat true for the current study, as older aged women were observed to be more susceptible to MetS in comparison to men. Although gender and age are non-modifiable risk factors for MetS with extensive controversies being postulated, modifiable risk factors such as lifestyle behaviors could provide more meaningful real-life interpretations. One such lifestyle behavior that could post substantial risk for people to be afflicted with MetS is sedentary work nature, which could be highly prevalent in the current study, as the study sample involved white-color workers in an academic institution whose occupational nature were mostly related to desk-jobs, thus predisposing to obesogenic effects.

#### Risk factors associated with MetS

The final regression model for the overall sample retained five risk factors to be associated with MetS, in which four variables (BMI, PA, hypertension and diabetes) showed statistical significance. When compared across gender, it was found that greater BMI, diabetes and hypertension increased the risk of MetS in women, while for men, although three factors were likely to increase the risk of MetS, only two (hypertension and diabetes) showed statistical significance. The regression model concluded that greater BMI in the overall sample (Wald value = 14.5) and in women (Wald value = 18.5) significantly predicted MetS. This finding was consistent with previous studies^26,42,66,67^. In obese individuals, free fatty acids and cytokines like tumor necrosis factor-alpha (TNF-α) are released by adipose cells. These substances block phosphatidylinositide-3-kinase-dependent signal transduction pathways, thus reducing glucose uptake in the liver and skeletal muscles^68,69^. As a consequence, pancreatic β-cells are forced to secrete excessive insulin. These conditions lead to hyperglycemia or diabetes^42^. As age advances, blood vessels tend to undergo gradual loosening of their elasticity, gaining increased resistance, and slowing of blood flow. With poor circulation, lipid is prone to pile up in the abdomen and release free fatty acids into the serum, causing greater insulin resistance and elevated serum triglycerides^70^. These physiological and biological processes, coupled with greater adiposity, predispose greater risk of MetS as observed in the current study, with the prevalence of MetS and its components being mostly increased with advancing age, and its associated factors, particularly diabetes, hypertension and hyperlipidemia being statistically significant in the final regression models.

The bulk of the literature had found that lifestyle behaviors such as PA, smoking and alcohol consumption escalates the risk of MetS^23,25,31,44,59^. In contrast to those investigations, the current study found a somewhat debatable finding on the associations between lifestyle behaviors and MetS. PA was significantly associated with MetS in the current study, consistent with previous reports^23,25,31^. However, moderate intensity PA was not a protective effect to MetS in the current study, similar to previous reports^71,72^. This could be attributed to the fact that moderate intensity PA may not suffice to accelerate metabolism and burn calories in a sample of individuals whose work is highly sedentary in nature. The other lifestyle behaviors, particularly smoking and alcohol consumption showed a peculiar pattern of association in the current study. Alcohol consumption which was significantly associated with MetS in the overall sample and in women at the univariate level showed a reversed observation across final regression models (not statistically significant for the overall sample and eliminated as a predictor for MetS in women). These findings contradict available literature^23,25,31^.

## Limitations

The cross-sectional nature of this study could not establish causal relationships. The relatively small sample size from a single center, coupled with non-representative demographics of the study population (such as majority being women) may limit the generalizability of the study findings, thus extrapolation of the study findings to a nationally representative estimate could not be established. Concurrently, the size of the sample in the current study may have increased the possibility of type II error in the current analysis. For example, alcohol drinking in the overall sample and greater BMI in men may have achieved statistical significance in association with MetS with a larger sample size (p = 0.079 and p = 0.058 respectively). The relatively wide confidence intervals (CIs) gap observed in certain variables, such as for the association between alcohol drinking and MetS suggests weak relationships, hence was not sufficiently powered enough to actually predict MetS in the current sample. As smoking and alcohol consumption were self-reported measures in the current study, the findings could be attributed to social-desirability bias. Such circumstances could be highly possible, as Malaysia, a country being shaped with cultural and religious norms, roles and behaviors may have predisposed respondents in the current sample to under-report alcohol consumption behaviors due to the apprehended harsh social etiquette issues or stigmatization within communities. This could be the reason on the relatively weak association of alcohol consumption and MetS in women at the univariate level, and its elimination or non-significance at the multivariate model. Similar under-reporting may have been attributed to smoking behaviors.

It should be noted that biochemical or physical examination data obtained during health screening sessions may be inconsistent with self-reported medical history by the respondents. For example, during examination, newly diagnosed hypertension or diabetes could have been reported, causing raised prevalence rates based on onsite blood pressure or blood glucose measurement as compared to self-reported medical history. In contrast, respondents who had their medical conditions controlled via compliance to anti-hypertensives, oral hypoglycemic agents or behavioral interventions would have observed reduced prevalence rates of hypertension, diabetes or hyperlipidemia at the time of study as compared to self-reported medical history. Succinctly, differences between baseline clinical parameters and MetS definition may pose inconsistencies as well. One of the definitions of MetS using the JIS criteria as adopted in this study was systolic BP ≥ 130 mmHg, and diastolic BP ≥ 85 mmHg. However, based on Malaysian Clinical Practice Guidelines, hypertension is defined as systolic BP ≥ 140 mmHg and diastolic BP ≥ 90 mmHg^73^. Despite these inconsistencies, the current study maintained self-reported medical history as independent variables, and not variables that defined the dependent variable (MetS) based on biochemical or physical examination data. This offsets redundancy of variable analytical procedures, which may escalate effect sizes with wide confidence interval gaps, resulting in risk for error to the study results.

## Conclusion

The overall prevalence of MetS in this sample was 20.6%. Older aged people were more likely to have MetS. Factors significantly associated with MetS in the overall sample were greater BMI, hypertension, diabetes and physical activity of moderate intensity. Gender-specific-risks regression models found that diabetes and hypertension were significantly associated with MetS in men, while greater BMI, diabetes and hyperlipidemia were significantly associated with MetS in women.

The results of the current study may have significant theoretical and practical implications within the academic settings. Operational definitions for components of MetS should be normalized with the national clinical practice guidelines after an expert panel review for the Malaysian population to halt inconsistencies of reported prevalence rates.

Based on gender specific prevalence trends of MetS, the current study may suggest post-menopausal age as a risk factor for MetS. Previous study from India have confirmed such associations^74^. However, as menopausal age of women (ranges between 40 and 55 years) was not confirmed, and given the cross-sectional nature of this investigation, the current study was not powered to establish its causality with MetS. This postulation could direct future studies for further exploration. The distinct gender-specific patterns of MetS risk factors are likely due to complex interactions of lifestyle, physiological, psychological and cultural influences. Such attributes are closely related to the nature of academics’ work environment which are mostly sedentary, multi-tasking and stressful. It would be worthwhile to note that treating individual risk components may be less useful to control MetS, but improvement of lifestyle behaviors and health interventions to identify high-risk employees would reduce the prevalence of MetS.

The findings of the current study catalyze the need to initiate employer-based interventions across the Malaysian academic settings. Workplace health promotion activities such as on-site diet and exercise programs should be executed more rigorously. Office of deaneries and departmental managers should actively plan and execute in-service health screening concerning MetS amongst their employees for physical and mental health monitoring.
